# TAK1 Regulates Myocardial Response to Pathological Stress via NFAT, NFκB, and Bnip3 Pathways

**DOI:** 10.1038/srep16626

**Published:** 2015-11-13

**Authors:** Lei Li, Yi Chen, Jing Li, Haifeng Yin, Xiaoyun Guo, Jessica Doan, Jeffery D. Molkentin, Qinghang Liu

**Affiliations:** 1 Department of Physiology and Biophysics, University of Washington, Seattle, WA 98195, USA; 2 Howard Hughes Medical Institute, Department of Pediatrics, Cincinnati Children’s Hospital Medical Center, Cincinnati, OH 45229, USA

## Abstract

TAK1 (TGFβ-activated kinase-1) signaling is essential in regulating a number of important biological functions, including innate immunity, inflammatory response, cell growth and differentiation, and myocardial homeostasis. The precise role of TAK1 in the adult heart under pathological conditions remains largely unknown. Importantly, we observed that TAK1 is upregulated during compensatory hypertrophy but downregulated in end-stage heart failure. Here we generated transgenic mice with inducible expression of an active TAK1 mutant (TAK1ΔN) in the adult heart. TAK1ΔN transgenic mice developed greater cardiac hypertrophy compared with control mice after transverse aortic constriction (TAC), which was largely blocked by ablation of calcineurin Aβ. Expression of TAK1ΔN also promoted NFAT (nuclear factor of activated T-cells) transcriptional activity in luciferase reporter mice at baseline, which was further enhanced after TAC. Our results revealed that activation of TAK1 promoted adaptive cardiac hypertrophy through a cross-talk between calcineurin-NFAT and IKK-NFκB pathways. More significantly, adult-onset inducible expression of TAK1ΔN protected the myocardium from adverse remodeling and heart failure after myocardial infarction or long-term pressure overload, by preventing cardiac cell death and fibrosis. Mechanistically, TAK1 exerts its cardioprotective effect through activation of NFAT/NFκB, downregulation of Bnip3, and inhibition of cardiac cell death.

Cardiac hypertrophy commonly occurs in response to hemodynamic stress, acute myocardial injury/infection, or genetic mutations in genes encoding sarcomeric proteins^1^. This process is partially adaptive and temporarily preserves pump function, but prolongation of this state often transits into dilated cardiomyopathy and heart failure^2^, highlighting the need for differentiating adaptive and maladaptive features of this process. Delineation of the signaling networks that control adaptive versus maladaptive cardiac responses is essential for the eventual elucidation of molecular mechanisms underlying the transition from cardiac hypertrophy to failure. Neural, humoral, and intrinsic hypertrophic stimuli directly activate membrane-bound receptors that in turn activate intracellular signaling pathways, including the mitogen-activated protein kinase (MAPK) cascade, calcineurin-nuclear factor of activated T-cells (NFAT), Insulin-like growth factor 1 (IGF-1)-phosphoinositide 3-kinase (PI_3_K)-Akt, and many others^3^,^4^,^5^. These intracellular signaling cascades then modulate transcriptional regulatory proteins, altering gene expression to promote hypertrophic growth of the heart. Transcriptional factors, such as NFATs, MEF2 (myocyte enhancer factor-2), GATA4/6 (GATA binding protein 4/6), NFκB (nuclear factor kappa-light-chain-enhancer of activated B cells), which are directly activated by cytoplasmic signaling effectors, mediate hypertrophic gene expression in cardiac myocytes^3^,^6^, Importantly, we recently showed that certain transcriptional signaling pathways, such as NFAT and NFκB, may interact to coordinate hypertrophic programming^7^.

Our previous study identified a novel signaling molecule, TAK1 (TGFβ-activated kinase 1), as a key regulator of the hypertrophic signaling network in cardiomyocytes *in vitro*^8^. TAK1 is an upstream member of the MAPK superfamily, which has been shown to function as a pivotal integrator of membrane-bound signals elicited by inflammatory cytokines (e.g., TNFα, TGFβ, IL-1, IL-8, RANKL), innate immunity signals, and bone morphogenetic proteins^9^,^10^. Importantly, TAK1 is upregulated in the mouse heart following pathological stress such as pressure overload and myocardial infarction, as well as in diseased human myocardium^11^,^12^,^13^. Previous studies by us and others suggest that TAK1 may play an important role in regulating cardiac hypertrophy and remodeling^8^,^11^,^13^. For example, transgenic expression of an active TAK1 mutant in neonatal mice induced cardiac hypertrophy, heart failure, and early lethality before 15 days of age^8^. However, the functional effects of TAK1 activation in the adult heart have not been investigated. Moreover, molecular mechanisms underlying TAK1-mediated cardiac hypertrophic response *in vivo* and the potential crosstalk between TAK1 and other hypertrophic signaling pathways are largely unknown. For instance, it has been shown that TAK1 activates several signaling pathways in non-myocytes, including JNK/p38 MAPK and IκB kinase (IKK)-NFκB, and recently the calcineurin-NFAT signaling^8^. However, the relative contributions of these signaling effectors to adaptive and maladaptive cardiac response remain unclear.

In addition to the role in regulating cardiac hypertrophy, our recent study revealed an important function for TAK1 in promoting myocardial survival and homeostasis by using cardiac-specific TAK1 knockout mice^14^. Indeed, cardiac-specific deletion of TAK1 led to adverse remodeling and heart failure, which were associated with spontaneous apoptotic and necroptotic cell death of cardiac myocytes. Consistent with our findings, ablation of TAK1 in non-cardiac tissues including skin, intestine, and liver, resulted in spontaneous cell death, inflammation, and fibrosis^15^,^16^,^17^. However, whether TAK1 activation is sufficient to confer cardioprotection following pathological stress *in vivo* has not been investigated. In this study, we generated an inducible cardiac-specific TAK1 transgenic mouse model to investigate its role in regulating cardiac hypertrophy and heart failure propensity in the adult mice *in vivo*. We provide evidence that activation of TAK1 in the adult heart promotes adaptive cardiac hypertrophy in response to pressure overload. Our results demonstrated that TAK1 regulates cardiac hypertrophy through a crosstalk between NFAT and NFκB transcriptional signaling pathways. Importantly, adult-onset inducible expression of TAK1 in transgenic mice protected the myocardium from adverse remodeling and heart failure after myocardial infarction or long-term pressure overload, by preventing myocardial cell death and fibrosis. Mechanistically, we determined that the cardio-protective effect of TAK1 signaling involves coordinated activation of NFAT/NFκB-mediated prosurvival pathways and downregulation of Bnip3 expression in the heart.

## Methods

An expanded Methods section is available as Supplemental Information.

### Animal models

A tetracycline-responsive binary α-MHC transgene system was used to allow temporally regulated expression of TAK1ΔN in cardiomyocytes of the heart^18^. The NFAT-luciferase reporter transgenic mouse and CnAβ−/− mouse have been described previously^19^,^20^. Experimental procedures with animals were approved by the Institutional Animal Care and Use Committee of University of Washington and all studies were carried out in accordance with the approved guidelines.

### Echocardiography, TAC, and MI

Echocardiography was performed with a VisualSonics Vevo 2100 imaging system as described^14^. The surgical procedure for transverse aortic constriction (TAC) and myocardial infarction (MI) has been described previously^7^,^21^.

### Luciferase reporter assays, histological analysis, cell size measurement, and TUNEL

Luciferase reporter assays from mouse hearts were performed as described^7^,^19^. Fibrosis was detected with Masson’s Trichrome staining on paraffin sections as described previously^14^. Cell surface area measurements and assessment of TUNEL from paraffin sections were performed as described^14^,^22^.

### Cell culture, adenoviral infection, cell death analysis, and Western blotting

Primary neonatal rat cardiomyocytes were prepared as previously described^7^. HL-1 cardiac myocyte cell line was kindly provided by Dr. Claycomb (Louisiana State University Health Sciences Center)^23^. AdshBNIP3 was kindly provided by Dr. Lorrie Kirshenbaum (University of Manitoba). Cell death was also measured using a Cell Meter Apoptotic and Necrotic Detection kit (ATT Bioquest, Sunnyvale, CA)^14^. Protein extraction from mouse heart or cultured cardiomyocytes and subsequent Western blotting were performed as described^14^,^21^.

### Statistics

Exact testing (Wilcoxan Rank-sum or Kruskal-Wallis test) was used for studies with small sample sizes. Data with normal distribution were also evaluated by one-way ANOVA with the Bonferroni’s post hoc test or repeated measures ANOVA. The log-rank test was used for the comparison of survival data. *P *< 0.05 was considered statistically significant.

## Results

### Generation of cardiac-specific inducible TAK1ΔN transgenic mice

**Figure 1:**
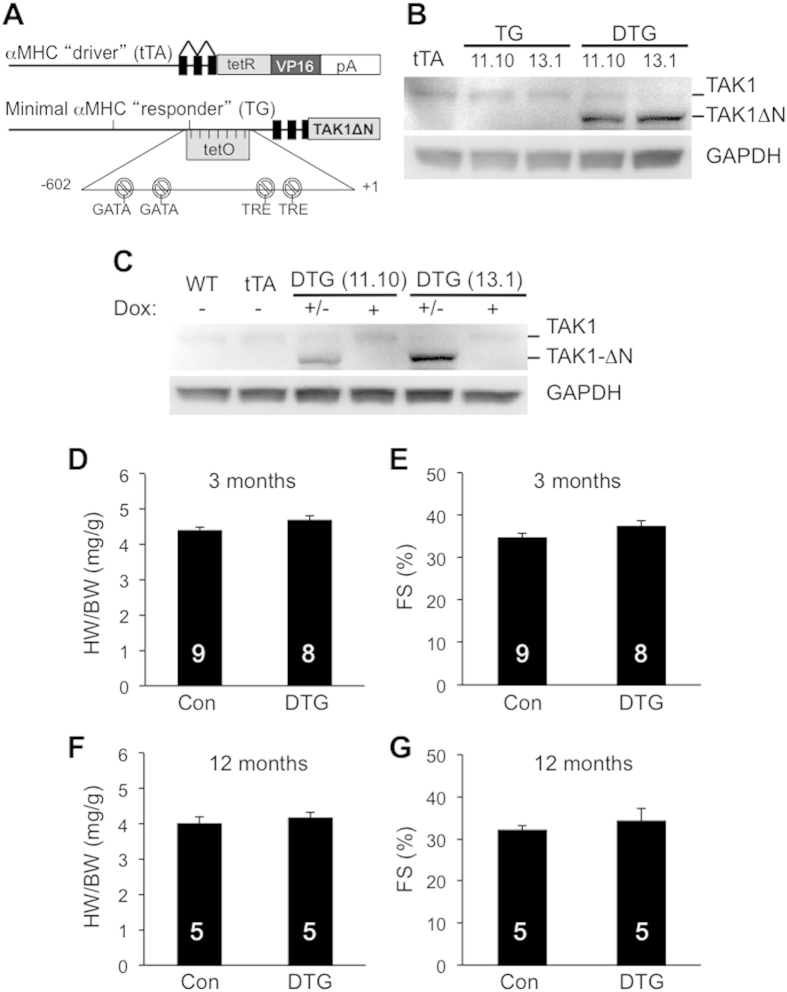
Characterization of the inducible cardiac-specific TAK1ΔN transgenic mice at baseline. **(A**) Schematic of the bitransgenic inducible expression system used to regulate TAK1ΔN expression in the mouse heart. (**B**) Western blot for TAK1 and glyceraldehyde 3-phosphate dehydrogenase (GAPDH) in cardiac extracts from mice of the indicated genotypes at 3 months of age (off Dox). 2 transgenic lines (11.10 and 13.1) were shown. (**C**) Western blot for TAK1 protein in the hearts of the indicated mice on Dox (+), off Dox (−), or breeding on Dox until weaning, and then off (+/−). (**D,E**) Heart weight/body weight ratio (HW/BW) and echocardiographic analysis of ventricular fractional shortening (FS) in TAK1ΔN transgenic line 11.10 at 3 months of age. (**F**,**G**) HW/BW and FS in TAK1ΔN transgenic line 11.10 at 12 months of age. The number of mice analyzed is shown in the bars of each panel.

Recent studies by us and others showed that TAK1 expression is upregulated in the heart following pressure overload or myocardial infarction^11^,^12^,^13^,^14^. To examine the physiological relevance of TAK1 upregulation *in vivo*, we modeled this effect by generating transgenic mice that permit inducible expression of the constitutively active TAK1ΔN in the adult heart. Heart-specific and -inducible expression was achieved with a binary α-MHC promoter-based transgene strategy (Fig. 1A)^18^. A modified α-MHC promoter was used as the responder transgene to promote TAK1ΔN expression when crossed with transgenic mice containing the α-MHC promoter-driven tetracycline transactivator (tTA) protein in the absence of doxycycline (Dox; Fig. 1A). TAK1ΔN transgenic lines were originally generated on FVB/N background, which were then backcrossed to C57Bl/6 for at least 6 generations. Two independent responder transgenic lines were produced (11.10 and 13.1) that each permitted expression of TAK1ΔN in the heart only in the presence of the driver transgene encoding tTA (double transgenic [DTG]) when Dox is absent (Fig. 1B). Wild-type, TAK1ΔN TG, and tTA TG mice showed no TAK1ΔN expression, which are used as control mice. To confirm inducible regulation by Dox, we observed that administration of Dox completely eliminated TAK1ΔN expression in DTG mice in both lines (Fig. 1C). For subsequent experiments, mice were bred with Dox treatment to block transgenic expression during embryonic and postnatal development, followed by removal of Dox at weaning to selectively permit transgene expression in the adult mice (Fig. 1C). In contrast to a previously study showing that conventional transgenic expression of TAK1ΔN in neonatal mouse caused early lethality^11^, inducible TAK1ΔN expression in the adult heart showed no signs of hypertrophy or changes in cardiac performance at 3 and 12 months of age (Fig. 1D through 1G). These results indicate that activation of TAK1 at early or late stages of cardiac development produces distinct cardiac phenotypes. The use of the inducible transgenic system thus bypasses developmental lethality observed with the conventional transgenic strategy and allows for the subsequent functional assessment of TAK1 in the adult mice as described below.

### TAK1ΔN expression promotes cardiac hypertrophy following pressure overload through the calcineurin-NFAT pathway *in vivo*

**Figure 2:**
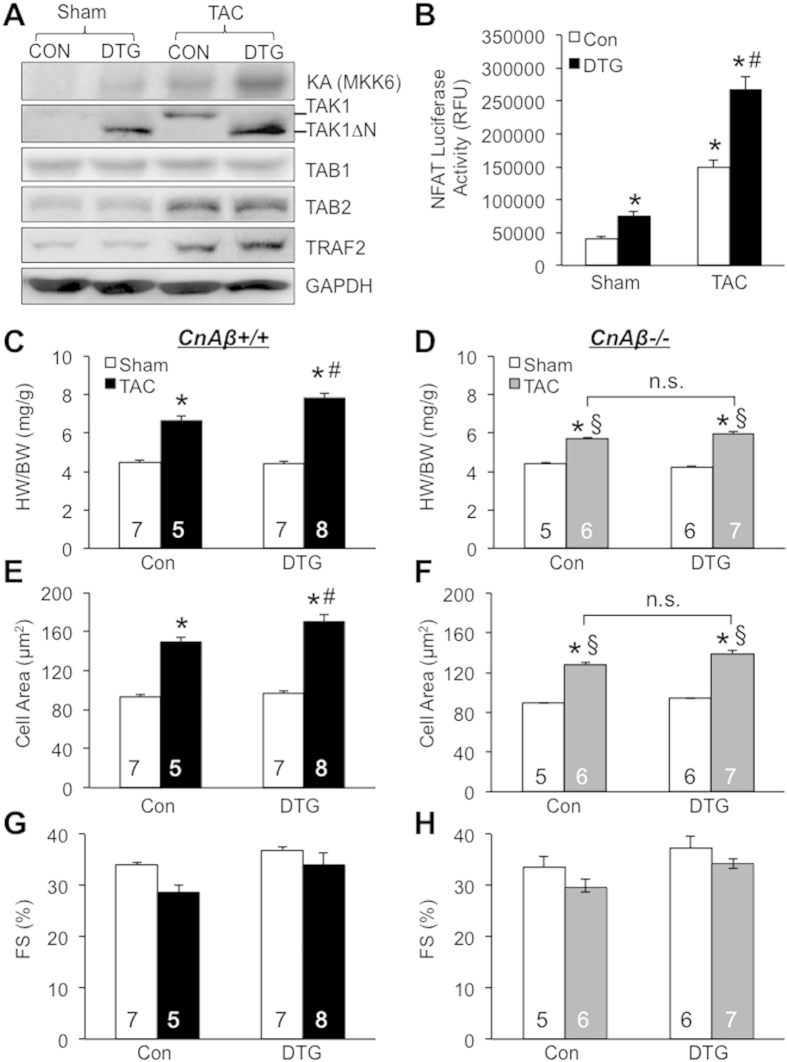
TAK1ΔN expression promotes cardiac hypertrophy following pressure overload through a calcineurin-dependent mechanism. (**A**) Autography of TAK1 kinase assay (KA) for MKK6 phosphorylation and Western blots for the indicated proteins from cardiac extracts of control and DTG mice subjected to TAC or sham procedure for 2 weeks. (**B**) Measurement of NFAT luciferase activity from control and DTG mice containing the NFAT luciferase reporter transgene after 2 weeks of TAC or sham procedure. *P < 0.05 versus Con Sham. ^#^*P *< 0.05 versus Con TAC. (**C**,**D**) Assessment of HW/BW from the indicated mice in the CnAβ+/+ (**C**) or CnAβ−/− (**D**) background subjected to TAC or sham procedure for 2 weeks. *P < 0.05 versus Sham. ^#^*P *< 0.05 versus Con TAC. ^§^*P *<  0.05 versus CnAβ+/+ Con TAC or CnAβ+/+ DTG TAC. (**E**,**F**) Myocyte surface area from cardiac sections of the mice indicated in C and D. Surface areas of 500 cells per mouse were measured in random fields. *P < 0.05 versus Sham. ^#^*P *<  0.05 versus Con TAC. ^§^*P *< 0.05 versus CnAβ+/+ Con TAC or CnAβ+/+ DTG TAC. (**G**,**H**) Cardiac fractional shortening (FS) of the mice indicated in C and D. n.s. denotes non-significance.

Because TAK1 is strongly induced by pressure overload^11^,^12^,^13^,^14^, we predict that TAC (transverse aortic constriction) stimulation would promote molecular coupling of the TAK1 signaling and cardiac hypertrophic response. We detected an increase in TAK1 kinase activity as well as protein expression in cardiac extracts after pressure overload by TAC (Fig. 2A). In addition, higher TAK1 kinase activity was detected in DTG mice after TAC compared with control mice. Importantly, two other signaling molecules in the TAK1 signaling pathway, TAB2 (TAK1 binding protein 2) and TRAF2 (TNF receptor-associated factor 2), were also upregulated (Fig. 2A). TAB2 is a key component of the TAK1 signaling complex acting as an adaptor protein that targets TAK1 to downstream effectors, such as RCAN1-calcineurin-NFAT^8^. TRAF2 is signaling molecule that links membrane receptors to the TAK1 complex, and it also mediates TAK1 polyubiquitination that is required for TAK1 activation and the recruitment of downstream signaling effectors^24^,^25^. Next, to directly examine the effect of TAK1 on cardiac hypertrophic signaling *in vivo*, we crossed the TAK1ΔN DTG mice with the NFAT-luciferase reporter mice^19^. TAK1ΔN DTG mice showed a mild but significant increase in NFAT-luciferase activity compared with control mice at baseline, which was significantly enhanced after 2 weeks of TAC (Fig. 2B). These data indicate that TAK1 activation promotes NFAT signaling in response to pressure overload *in vivo*.

To determine the effect of TAK1 activation on cardiac hypertrophy following pressure overload, DTG and control mice were subjected to TAC for 2 weeks. DTG mice showed significantly greater cardiac hypertrophy compared with control mice, as indicated by increased heart weight/body weight ratio and cardiomyocyte surface area (Fig. 2C,E). However, ventricular performance in DTG mice was not negatively affected after 2 weeks of TAC stimulation (Fig. 2G). Therefore, activation of TAK1 in the adult heart promoted cardiac hypertrophy with preserved ventricular function in response to pressure overload. As an important control, DTG mice were treated with Dox to block TAK1ΔN expression, which showed similar hypertrophic response as control mice after 2 weeks of TAC (Supplemental Figure 1). Our previous *in vitro* study showed that TAK1 induced cardiomyocyte hypertrophic growth through a calcineurin-dependent mechanism^8^,^26^. To verify this important observation *in vivo*, we crossed DTG mice into the calcineurin Aβ null (CnAβ−/−) background^27^. Importantly, DTG mice lacking CnAβ failed to show enhanced cardiac hypertrophy after 2 weeks of TAC compared with littermate controls in the same CnAβ−/− background (Fig. 2D,F). Ablation of CnAβ had no significant effects on ventricular contractile function in DTG and control mice (Fig. 2H). These results suggest that TAK1ΔN expression promotes cardiac hypertrophy following pressure overload through the calcineurin-NFAT pathway *in vivo*.

### TAK1 activates hypertrophic signaling through a crosstalk between NFAT and NFκB signaling pathways

**Figure 3:**
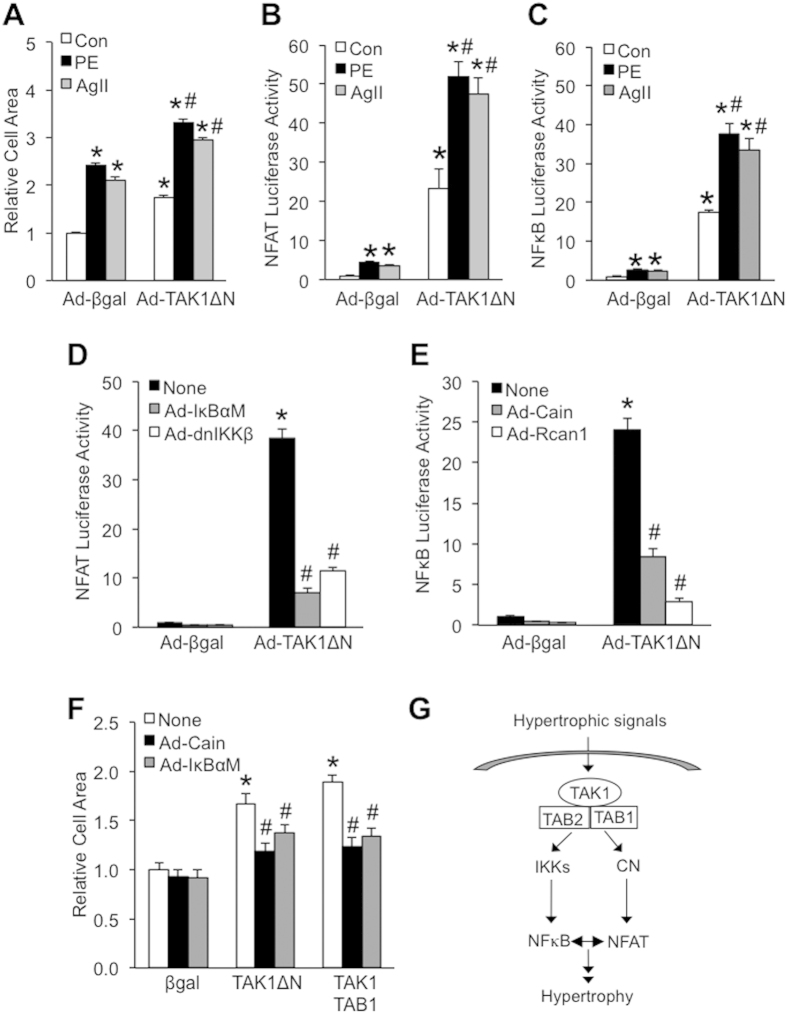
TAK1 regulates cardiomyocyte hypertrophy through NFAT and NFκB. (**A**) Surface areas of cardiomyocytes infected with β-gal or TAK1ΔN adenoviruses, followed by stimulation with vehicle control (Con), phenylephrine (PE, 50 μmol/L), or angiotensin II (AngII, 100 nmol/L) for 24 h. *P < 0.01 versus Con Ad-βgal; ^#^*P *< 0.05 versus Con Ad-TAK1ΔN. (**B**,**C**) NFAT-luciferase and NFκB-luciferase activity in cardiomyocytes infected with adenoviruses expressing NFAT-luciferase reporter along with β-gal or TAK1ΔN, followed by stimulation with vehicle control, PE, or AngII. *P < 0.05 versus Con Ad-β-gal; ^#^*P *< 0.05 versus Ad-TAK1ΔN. (**D**) NFAT-luciferase activity in cardiomyocytes infected with adenoviruses expressing NFAT-luciferase reporter along with β-gal, TAK1ΔN, IκBαM, or dnIKKβ. *P < 0.01 versus None Ad-βgal; ^#^*P *< 0.05 versus None Ad-TAK1ΔN. (**E**) NFκB-luciferase activity in cardiomyocytes infected with adenoviruses expressing NFκB-luciferase reporter along with β-gal, TAK1ΔN, Cain or Rcan1. *P < 0.01 versus None Ad-βgal; ^#^*P *< 0.05 versus None Ad-TAK1ΔN. (**F**) Surface areas of cardiomyocytes infected with βgal, TAK1ΔN, TAK1 plus TAB1 adenoviruses along with Cain or IκBαM. *P < 0.05 versus None Ad-βgal; ^#^*P *< 0.05 versus None Ad-TAK1ΔN or None TAK1 plus TAB1. (**G**) Schematic representation of the TAK1-activated hypertrophic signaling pathway.

To determine if activation of TAK1 enhances hypertrophic response in cardiomyocytes, neonatal cardiomyocytes were infected with adenoviral vectors encoding TAK1ΔN or β-galactosidase (β-gal) as a control, followed by stimulation with hypertrophic agonists phenylephrine (PE) or angiotensin II (AngII). Indeed, overexpression of TAK1ΔN further increased PE- and AngII- induced hypertrophic growth of cardiomyocytes (Fig. 3A). Both calcineurin-NFAT and IKK-NFκB signaling pathways has been implicated as critical regulators of cardiomyocyte hypertrophy^7^,^26^,^28^. Although our data established a role for TAK1 in regulating NAFT signaling, its role in NFκB signaling in cardiomyocytes has not been examined. To this end, we determined that TAK1ΔN expression was sufficient to induce NFκB luciferase activity in cardiomyocytes, which was blocked by co-expression of the NFκB supersuppressor IκBα S32/36A mutant (IκBαM) or dominant negative IKKβ (dnIKKβ) (Supplemental Figure 2A). Furthermore, hypertrophic agonists-induced NFκB transcriptional activity was largely blocked by overexpression of the dominant negative TAK1-KW, suggesting an essential role for TAK1 in regulating NFκB signaling in cardiomyocytes (Supplemental Figure 2B). Similarly, we previously showed that TAK1 is also essential for the activation of NFAT signaling^8^. Next, we examined how TAK1 affects NFAT and NFκB signaling in neonatal cardiomyocytes following stimulation with hypertrophic agonists. Cardiomyocytes were infected with adenoviruses encoding NFAT or NFκB dependent luciferase reporter cassette, along with TAK1ΔN or β-gal control adenoviruses, followed by treatment with vehicle, PE, or AngII. TAK1ΔN overexpression further enhanced hypertrophic agonist-induced NFAT and NFκB transcriptional activity (Fig. 3B,C). These results suggest that activation of TAK1 promotes both NFAT and NFκB signaling in cardiomyocytes.

Based on the observation that TAK1 activates both NFAT and NFκB signaling in cardiomyocytes, we hypothesized that these two hypertrophic signaling pathways may crosstalk with one another as part of the TAK1 signaling network to regulate hypertrophic response. Intriguingly, TAK1-induced NFAT luciferase activity was significantly reduced by inhibition of NFκB signaling with IκBαM or dnIKKβ (Fig. 3D). On the other hand, TAK1-induced NFκB luciferase activity was also diminished by inhibition of NFAT signaling with calcineurin inhibitors Cain or Rcan1 (Fig. 3E). These data suggest a crosstalk mechanism whereby NFAT and NFκB interdependently regulate one another’s transcriptional activity in TAK1-mediated hypertrophic signaling. Moreover, hypertrophic growth induced by TAK1 activation (with TAKΔN or TAK1 plus its activator TAB1) depends on both NFAT and NFκB signaling, since this effect was blocked by either Cain or IκBαM (Fig. 3F). Taken together, our results suggest that TAK1 regulates hypertrophic growth through a crosstalk between NAFT and NFκB signaling pathways (Fig. 3G).

### Expression of TAKΔN downregulates Bnip3 in cardiomyocytes through activation of NFκB

**Figure 4:**
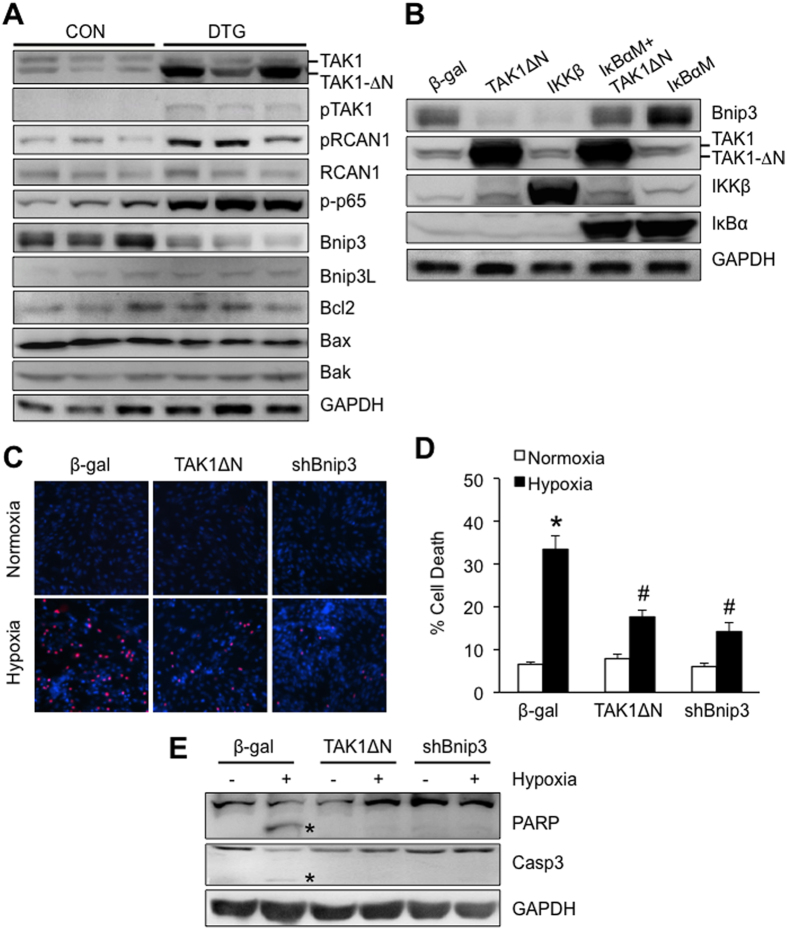
TAKΔN overexpression downregulates Bnip3 in cardiomyocytes through activation of NFκB. (**A**) Western blot for the indicated proteins in cardiac extracts from control and DTG mice at 3 months of age. (**B**) Western blots for the indicated proteins in cellular extracts from HL-1 cadiomyocytes infected with adenoviruses expressing β-gal, TAK1ΔN, IKKβ, or IκBαM. (**C**) TUNEL (red) and Hoechst staining of nuclei (blue) in HL-1 cardiomyocytes infected with adenoviruses expressing β-gal, TAK1ΔN, or Bnip3 shRNA, followed by normoxia or hypoxia for 24 h. (**D**) Quantification of cell death in HL-1 cardiomyocytes as indicated in C. *P < 0.01 versus β-gal Normoxia; ^#^*P *< 0.05 versus β-gal Hypoxia. (**E**) Western blot for PARP and caspase 3 in cellular extracts of HL-1 cardiomyocytes treated as described in C. Asterisk indicates cleaved form of PARP or caspase 3.

We previously showed that TAK1 regulates calcineurin-NFAT signaling through phosphorylation of Rcan1 (regulator of calcineurin 1) *in vitro*^8^. Consistent with this, here we observed an increase in Rcan1 phosphorylation at Ser136 in cardiac extracts from DTG mice, while no changes in Rcan1 expression were observed (Fig. 4A). In addition, phosphorylation of TAK1 and NFκB-p65 was also increased in DTG mice (Fig. 4A). Importantly, in DTG mice we observed a significant downregulation of Bnip3 (Bcl-2/adenovirus E1B 19 KDa protein-interacting protein 3), which has been implicated as a critical regulator of cardiac cell death and pathological remodeling^29^. No changes were detected in the expression levels of other bcl-2 family proteins including Bnip3L, Bcl-2, Bax, and Bak (Fig. 4A). It has been shown that NFκB negatively regulates Bnip3 expression in cardiomyocytes by transcriptional silencing^30^. We hypothesized that TAK1 may regulate Bnip3 expression through an NFκB dependent mechanism. To test this, HL-1 cardiomyocytes were infected with adenoviruses encoding TAKΔN along with the NFκB supersuppressor IκBαM. Overexpression of TAKΔN downregulated Bnip3 expression in cardiomyocytes (Fig. 4B). Similar effects were observed with overexpression of IKKβ, an upstream kinase for NFκB. Importantly, downregulation of Bnip3 by TAK1 was blocked by inhibition of NFκB with IκBαM (Fig. 4B). These data suggest that TAK1 negatively regulates Bnip3 expression through activation of NFκB. Bnip3 knockdown or dominant negative inhibition has been shown to inhibit hypoxic cardiomyocyte death^31^,^32^. This prompted us to examine whether TAK1 activation influences cell death in cardiomyocytes. Indeed, adenoviral overexpression of TAKΔN inhibited hypoxia-induced cell death and the cleavage of PARP and caspase 3 in HL-1 cardiomyocytes (Fig. 4C–E). Similar effects were observed in cells infected with an adenovirus encoding Bnip3 shRNA. Together, these data suggest that TAK1 activation promotes cell survival signaling through several mechanisms including activation of NFAT/NFκB pathways and downregulation of Bnip3.

### TAK1ΔN DTG mice are protected from chronic pressure overload-induced pathological cardiac remodeling and dysfunction

**Figure 5:**
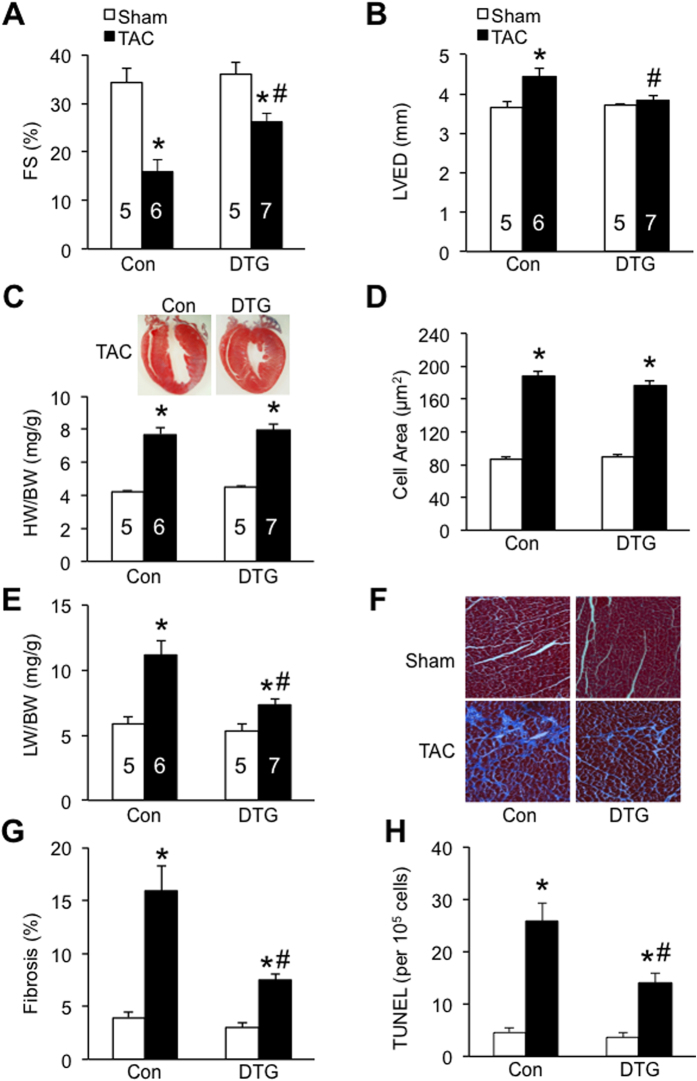
TAK1ΔN DTG mice are protected from chronic pressure overload-induced pathological cardiac remodeling and dysfunction. (**A**,**B**) Cardiac fractional shortening (FS) and left ventricular end-diastolic dimension (LVED) from control and DTG mice subjected to TAC or sham procedure for 8 weeks. *P < 0.05 versus corresponding Sham. ^#^*P *< 0.05 versus Con TAC. (**C**) Heart weight/body weight ratio (HW/BW) from control and DTG mice indicated in A. Masson trichrome-stained whole heart sections from control and DTG mice subjected to TAC are shown. (**D**) Myocyte surface area from cardiac sections of the mice indicated in A. (**E**) Lung weight/body weight ratio (LW/BW) from mice indicated in A. *P < 0.05 versus corresponding Sham. ^#^*P *< 0.05 versus Con TAC. (**F**) Masson trichrome-stained, paraffin-embedded cardiac sections from mice indicated in A. (**G**) Quantitation of cardiac fibrotic area from cardiac sections indicated in F. *P < 0.01 versus corresponding Sham. ^#^*P *< 0.05 versus Con TAC. (**H**) TUNEL-positive nuclei in cardiac sections. *P < 0.01 versus corresponding Sham. ^#^*P *< 0.05 versus Con TAC.

Next, we assessed the potential role of TAK1 in regulating cardiac remodeling and heart failure propensity following pathological stress. Control and DTG mice were subjected to long-term pressure overload by TAC for 8 weeks. Echocardiographic analysis of cardiac function showed ventricular contractile dysfunction and chamber dilation in control mice following chronic pressure overload (Fig. 5A,B), while DTG mice showed better contractile function and less ventricular dilation (Fig. 5A,B). Interestingly, in contrast to short term TAC stimulation, prolonged pressure overload with 8 weeks of TAC induced similar level of cardiac hypertrophy in DTG and control mice, as assessed by heart weight to body weight ratio as well as myocyte surface area (Fig. 5C,D). However, cardiac hypertrophy in DTG mice did not lead to ventricular dilation as seen in control mice (Fig. 5A–C), suggesting that TAK1 activation induces an adaptive hypertrophic response in the adult heart. Moreover, DTG mice showed less pulmonary congestion as measured by lung weight/body weight ratio and less myocardial fibrosis as assessed by Masson’s trichrome staining compared with control mice (Fig. 5E–G). A significant decrease in TUNEL (terminal deoxynucleotidyl transferase dUTP nick end labeling) positive cells and caspase 3 cleavage was also detected in cardiac sections of DTG mice (Fig. 5H and Supplemental Figure 3). These data collectively suggest that TAK1 activation in adult heart is cardioprotective by preventing pathological remodeling, functional decompensation, and heart failure progression following chronic pressure overload.

### TAK1 activation prevented cardiac cell death, pathological remodeling, and heart failure progression after myocardial infarction

**Figure 6:**
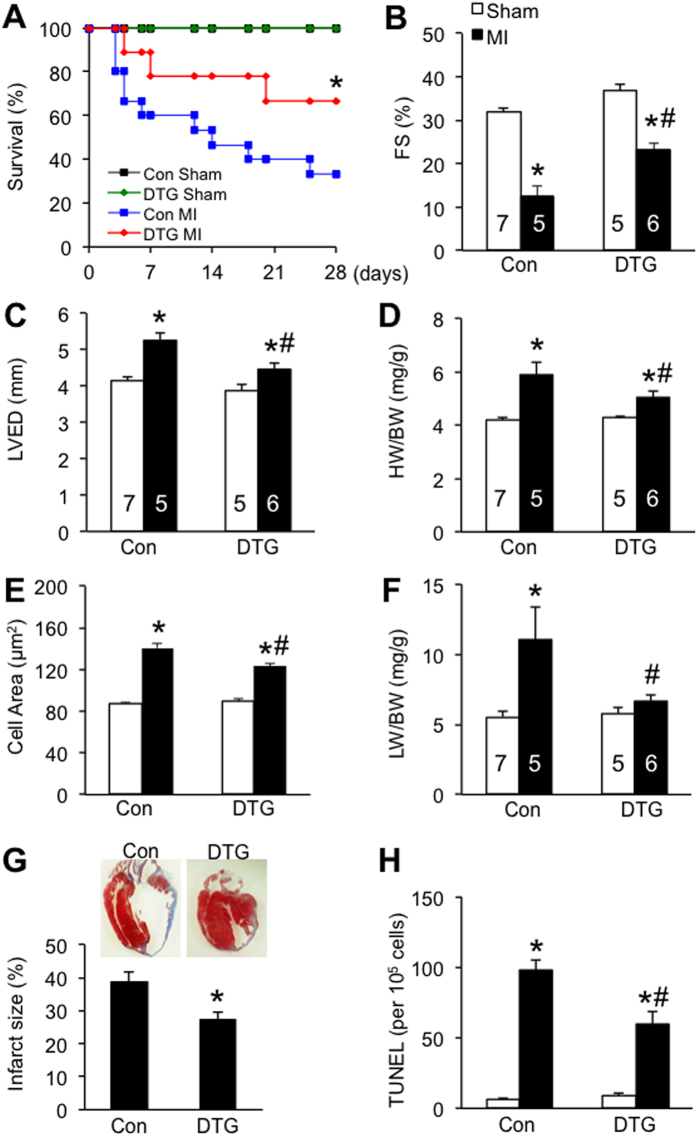
TAK1 activation prevented pathological remodeling and heart failure progression after myocardial infarction. (**A**) Survival rates of Con and DTG mice subjected to sham or MI surgical procedure for 4 weeks. *P < 0.05 versus Con MI. **(B**,**C**) Echocardiographic analysis of FS and LVED in Con and DTG mice subjected to sham or MI surgical procedure for 4 weeks. *P < 0.05 versus corresponding Sham. ^#^*P *< 0.05 versus Con MI. (**D,E**) HW/BW and cell surface area from mice indicated in B. *P < 0.05 versus corresponding Sham. ^#^*P *< 0.05 versus Con MI. (**F**) LW/BW from mice indicated in B. *P < 0.05 versus corresponding Sham. ^#^*P *< 0.05 versus Con MI. (**G**) Masson’s trichrome-stained histological cardiac sections and quantitation of infarct size from Con or DTG mice subjected to MI or a sham procedure for 4 weeks. *P < 0.05 versus Con. (**H**) TUNEL-positive nuclei in the cardiac sections from control and DTG mice. *P < 0.01 versus corresponding Sham. ^#^*P *< 0.05 versus Con MI.

Finally, we examined the role of TAK1 in another model of heart failure induced by myocardial infarction (MI). Control and DTG mice were subjected to MI or a sham procedure for up to 4 weeks. A significantly higher survival rate was observed in DTG mice compared to litter mate controls after 4 weeks of MI (Fig. 6A). Echocardiographic analysis showed better cardiac function with less ventricular dilation in DTG mice (Fig. 6B,C). Intriguingly, DTG mice displayed less secondary hypertrophy following MI compared with control mice (Fig. 6D,E). Diminished pulmonary congestion was also observed in DTG mice (Fig. 6F). Masson’s trichrome staining of cardiac sections showed smaller infarct size and less chamber dilation in DTG mice compared with control mices after 4 weeks of MI (Fig. 6G). Moreover, a significant decrease in TUNEL positive cells and cleaved caspase 3 was observed in DTG mice compared with control mice (Fig. 6H, Supplemental Figure 4). DTG mice also showed reduced plasma levels of HMGB1 (high-mobility group box 1), a biomarker of necrotic cell death and myocardial damage^14^, compared with control mice after acute MI of 24 h (Supplemental Figure 4). Taken together, these results indicate that TAK1 activation in the adult heart prevented cardiac cell death, pathological remodeling, and heart failure progression after myocardial infarction.

## Discussion

TAK1 signaling is critical in regulating a number of important biological processes, including immune response, inflammation, proliferation and differentiation, angiogenesis, and cardiomyocyte hypertrophic growth^8^,^11^,^13^,^33^. Our recent study using a cardiac-specific TAK1 knockout mouse model identified an essential role for TAK1 in regulating myocardial survival and remodeling^14^. In this study, we generated an inducible cardiac-specific TAKΔN transgenic mouse model to gain further insight into the physiological function and molecular regulation of TAK1 in the adult heart. We showed that activation of TAK1 in the adult heart induced an adaptive cardiac hypertrophic response through a crosstalk between calcineurin-NFAT and IKK-NFκB signaling pathways. Moreover, our data revealed a new cardioprotective role of TAK1 in the adult heart *in vivo*. Indeed, adult-onset inducible expression of TAK1 protected the heart from adverse remodeling and cardiac dysfunction following chronic pressure overload or myocardial infarction, by preventing cardiac cell death and fibrosis. Mechanistically, TAK1 coordinately activates NFAT/NFκB prosurvival pathways and downregulated Bnip3 expression in the heart. These results suggest that TAK1 plays a critical role in the adaptive myocardial response to pathological stress, by regulating cardiac hypertrophy, myocardial remodeling, and heart failure propensity.

In mice, the expression level of TAK1 is relatively high during early stages of cardiac development and gradually decreases towards adulthood^11^. It has been shown that TAK1 is upregulated in response to pathological stress such as pressure overload and myocardial infarction^11^,^12^. Importantly, we previously observed that TAK1 is upregulated during compensatory cardiac hypertrophy but downregulated during end-stage heart failure^14^. These data suggest that TAK1 may functions as a disease modifying kinase and upregulation of TAK1 may represent an important adaptive mechanism under pathological conditions. Our inducible TAKΔN transgenic mouse had no discernable phenotype at baseline up to 12 months old, but developed enhanced cardiac hypertrophy after pressure overload. TAK1 overexpression in the adult heart was likely without baseline phenotype given many levels of regulation imposed on this kinase. TAK1 is tightly regulated by post-translational modifications (e.g., ubquitination and phosphorylation) and physical interaction with other signaling modulators. For example, TRAF2 functions as an ubiquitin E3 ligase to mediate TAK1 polyubiquitination, which is critical for the recruitment of downstream signaling effectors^24^,^25^. TAB1 is a TAK1 binding protein that functions as an activator of TAK1 by promoting its auto-phosphorylation^34^. TAB2 is another TAK1 binding protein that acts as an adaptor protein linking TAK1 to downstream targets such as the Rcan1-calcineurin-NFAT signaling complex^8^. Importantly, both TRAF2 and TAB2 were upregulated in the heart by pressure overload, which will facilitate the molecular coupling between TAK1 and downstream hypertrophic signaling effectors. This may account for the enhanced cardiac hypertrophic response in TAK1ΔN transgenic mice after pressure overload.

In contrast to our observations in the adult mice using an inducible system, Zhang *et al.* showed that extensive expression of TAK1ΔN in neonatal mice led to hypertrophic cardiomyopathy, heart failure, and premature death within 2 weeks after birth^11^. In that study a conventional transgenic approach was used and a high level of TAK1ΔN expression was produced using the standard αMHC promoter. Similarly, forced expression of TAK1ΔN was sufficient to induce hypertrophic growth in neonatal cardiomyocytes. We speculate that the timing and extent of TAK1 expression is critical in determining its functional effects in the heart. Indeed, distinct phenotypes and functional consequences associated with neonatal versus adult transgenic expression systems have been reported. For example, inducible expression of Gαq protein in the adult heart failed to reproduce phenotypic features of cardiomyopathy caused by neonatal expression of Gαq driven by the αMHC promoter^35^. Also noteworthy is that inducible expression of active calcineurin in the adult heart only induced minimal hypertrophy, whereas conventional transgenic expression of calcineurin led to massive cardiac hypertrophy^36^. Here we showed that activation of TAK1 in the adult heart further enhanced cardiac hypertrophy after pressure overload by 2 weeks of TAC. Intriguingly, chronic pressure overload by 8 weeks of TAC induced similar hypertrophic response in control and DTG mice, probably due to more prominent pathological remodeling in control mice as compared to DTG mice in response to prolonged TAC stimulation, as indicated by increased fibrosis and cell death. Moreover, myocardial infarction, which triggers distinct hypertrophic regulatory mechanism from pressure overload, didn’t induce more hypertrophy in DTG mice than control mice, suggesting a role for TAK1 in adaptive but not maladaptive cardiac hypertrophy.

The molecular mechanisms underlying the role of TAK1 in regulating cardiac hypertrophy have not been investigated *in vivo*. We previously observed that overexpression of TAK1ΔN activated the calcineurin-NFAT signaling in cultured cardiomyocytes^8^. Here we extended this observation in our transgenic mice. Specifically, we crossed the TAK1ΔN transgenic mice into the CnAβ−/− background as a way to reduce total calcineurin activity in the heart^27^. Deletion of CnAβ attenuated the ability of TAK1 to promote cardiac hypertrophy upon pressure overload stimulation. Moreover, activation of TAK1 induced NFAT transcriptional activity in luciferase reporter mice in the basal state and after pressure overload, providing direct evidence that TAK1 activates calcineurin-NFAT signaling *in vivo*. We also demonstrated that TAK1 is both sufficient and necessary in activating another transcription factor, NFκB, in cardiomyocytes, which has also been implicated as a key regulator of cardiomyocyte hypertrophy^37^,^38^. Intriguingly, we showed that TAK1-induced NFAT transcriptional activity was partially blocked by inhibition of NFκB and *vice versa*, suggesting a crosstalk between these two transcriptional pathways as we recently described^7^. We speculate that coordinated activation of NFAT and NFκB by TAK1 may account for the enhanced hypertrophic signaling in the heart. However, TAK1 activation in the adult heart, which is associated with mild activation of NFAT and NFκB pathways, does not induce hypertrophy under basal conditions. It has been shown that activation of calcineurin in the adult heart leads to mild activation of NFAT, but no significant hypertrophy^36^. Moreover, activation of NFκB by forced expression of IKKβ induced dilated cardiomyopathy without cardiomyocyte hypertrophy^39^. These results suggest that NFAT or NFκB activation may not be sufficient to induce hypertrophic response in the adult heart under basal conditions. Additional signaling input is needed to trigger cardiac hypertrophy program *in vivo*, based on the observation that TAK1 activation promoted hypertrophic response following pathological stress, such as pressure overload. This further supports that notion that TAK1 functions as a stress response signaling pathway.

Whether the upregulation of TAK1 upon pathological stress is adaptive or maladaptive has not been directly addressed *in vivo*. Here we observed a prominent cardioprorective effect in TAK1ΔN mice after chronic pressure overload as well as myocardial infarction. We further determined that the cardioprotective effect of TAK1 involves coordinated activation of NFAT and NFκB pathways as well as downregualtion of Bnip3 expression in the heart. In addition to an essential role in signaling cardiac hypertrophy, it has been suggested that the calcineurin-NFAT pathway may represent a protective mechanism in myocardium. For example, genetic deletion of calcineurin Aβ predisposed the heart to acute ischemia-induced apoptosis and dysfunction, while overexpression of calcineurin prevented cardiac cell death and pathological remodeling after ischemia-reperfusion^20^,^40^. The transcription factor NFAT has been identified as a downstream effector of the protective effects of calcineurin in cardiomyocytes^41^. Similarly, the cardioprotective role of the NFκB pathway has been well established. For example, inhibition of NFκB by cardiac-specific expression of the non-phosphorylatable IκB mutant increased myocyte apoptosis following acute coronary occlusion^42^,^43^. On the other hand, activation of NFκB by forced expression of IKKβ largely blocked hypoxia-induced cell death and mitochondrial defects in cardiomyocytes^44^. Importantly, it has been shown that NFκB-mediated cell survival involves transcriptional silencing of the mitochondrial death gene Bnip3^30^. In the present study, we showed that activation of TAK1 downregulated Bnip3 expression in cardiomyocytes through an NFκB dependent mechanism. In line with this, we previously observed that Bnip3 was upregulated in TAK1-deficient mice^14^. It was shown that ablation of Bnip3 prevented adverse myocardial remodeling and cardiac dysfunction by inhibition of apoptosis following ischemia-reperfusion, whereas forced expression of Bnip3 promoted cardiac cell death, ventricular dilation, and systolic dysfunction^29^,^31^,^32^. Thus downregulation of Bnip3 may represent a critical mechanism for the cardioprotective effects of TAK1 in the heart. Our data showed that activation of TAK1 inhibited hypoxia-induced cell death in cardiomyocytes. Moreover, we previously observed that activation of TAK1 inhibited TNFα receptor-mediated cell death signaling^14^. Therefore, TAK1 may exert its cardioprotective effects through multiple mechanisms including activation of prosurvival NFAT/NFκB pathway, downregulation of Bnip3, and direct regulation of cell death signaling.

In summary, the highly interconnected signaling effector TAK1 mediates adaptive hypertrophy in response to pressure overload, through a crosstalk between calcineurin-NFAT and IKK-NFκB signaling pathways. Moreover, activation of TAK1 confers cardioprotection in the adult heart following pathological stress. This notion is consistent with our previous study showing that genetic or pharmacological inactivation of TAK1 in mice caused spontaneous myocyte death, adverse remodeling, and heart failure. Mechanistically, TAK1 functions as a nodal control point in regulating calcineurin-NFAT, IKK-NFκB, Bnip3, and cell death signaling in the heart. Our finding that TAK1 has a protective role in the heart following pathological stress suggests that components of the TAK1 signaling pathway may serve as potential diagnostic and therapeutic targets. Interventions designed to selectively activate the TAK1 signaling pathway may prove beneficial by preventing cardiac cell death, adverse remodeling, and heart failure progression.

## Additional Information

**How to cite this article**: Li, L. *et al.* TAK1 Regulates Myocardial Response to Pathological Stress via NFAT, NFκB, and Bnip3 Pathways. *Sci. Rep.* **5**, 16626; doi: 10.1038/srep16626 (2015).

## Supplementary Material

Supplementary Information
