# Supplementary material for: TAK1 Regulates Myocardial Response to Pathological Stress via NFAT, NFκB, and Bnip3 Pathways
Source: Sci Rep. 2015 Nov 13;5:16626. doi: 10.1038/srep16626 (PMC4643217; doi:10.1038/srep16626)
Supplement: Supplementary Information [file srep16626-s1.pdf]

TAK1 Regulates Myocardial Response to Pathological Stress via NFAT, NFκB, and Bnip3 Pathways

Lei Li[1,†], Yi Chen[1,†], Jing Li[1], Haifeng Yin[1], Xiaoyun Guo[1], Jessica Doan[1], Jeffery D. Molkentin[2], and Qinghang Liu[1,*]

SUPPLEMENTAL INFORMATION

Methods

Animal Models

A tetracycline-responsive binary α-MHC transgene system was used to allow temporally regulated expression of TAK1ΔN in cardiomyocytes of the heart.[18] TAK1ΔN transgenic mice were originally generated on FVB/N background and were subsequently backcrossed into C57Bl/6 for at least 6 generations. Doxycycline (Dox) was administered in food with a special diet formulated by Purina (625 mg/kg in pellets). In experiments that required TAK1ΔN protein induction, Dox was removed from the food at weaning, resulting in induced expression of TAK1ΔN a few weeks later in young adulthood. The NFAT-luciferase reporter transgenic mouse and CnAβ-/- mouse have been described previously[19,20] and were crossed with the TAK1ΔN transgenic mouse. Experimental procedures with animals were approved by the Institutional Animal Care and Use Committee of University of Washington and all studies were carried out in accordance with the approved guidelines.

Echocardiography, TAC, and MI

For echocardiography, mice were anesthetized with 2% isoflurane by inhalation and scanning was performed with a VisualSonics Vevo 2100 imaging system as described previously.[14] M-mode left ventricular dimensions were averaged from 3-5 beats. Fractional shortening (FS) was

calculated using left ventricle dimensions in end of systole and diastole (LVES and LVED, respectively): FS = [(LVED - LVES)/LVED] x 100 (%).[14] Transverse aortic constriction (TAC) was performed to produce cardiac pressure overload in mice using a 27-gauge needle as previously described.[7] Sham-operated mice underwent the same procedure without aortic constriction. Pressure gradients (PG; mm Hg) across the aortic constriction were calculated from the peak blood velocity (*Vmax*) (m/s) (PG = 4 x $Vmax^2$) measured by Doppler, which was equivalent in all groups of TAC stimulated mice. The surgical procedure for myocardial infarction (MI) injury in the mouse has been described previously.[21] Mice were sacrificed by CO2 asphyxiation, and hearts were analyzed using 2% triphenyltetrazolium chloride in saline and 2% Evan's blue dye infusion to identify infarct area.[21]

Histological analysis, cell size measurement, and TUNEL

For histological analysis, mouse hearts were fixed in 10% formalin/phosphate-buffered saline and dehydrated for paraffin embedding. Fibrosis was detected with Masson's Trichrome staining on paraffin sections. Blue collagen staining was quantified using MetaMorph 6.1 software as described previously.[14] For cell surface area measurements, membranes were stained with TRITC- or FITC- labeled lectin from Triticum vulgaris (Sigma), and nuclei were labeled with TO-PRO 3 iodine (Molecular Probes, Carlsbad, CA). Cellular areas were quantified with ImageJ 1.33 software (Scion Corp., Frederick, MD). Assessment of TUNEL from paraffin sections was performed with an ApopTag Peroxidase In Situ Apoptosis Detection Kit (Millipore) according to the manufacturer's instructions or a TMR Red In Situ Death Detection Kit (Roche Diagnostics) as described in detail previously.[14,22]

Luciferase reporter assays in mouse hearts

Luciferase reporter assays from mouse hearts were performed as described.[7] Briefly, hearts were removed from NFAT-luciferase transgenic mice, some of which were crossed with

TAK1ΔN transgenic mice. Hearts were homogenized in 1 ml luciferase assay buffer (100 mM KH_2PO4, pH 7.8, 0.5% Nonidet P-40, and 1 mM DTT). Homogenates were centrifuged at 3,000 *g* for 10 min at 4°C and the supernatants assayed for luciferase activity as described.[7,19]

Cell culture, adenoviral infection, and cell death analysis

Primary neonatal rat cardiomyocytes were prepared from hearts of 1- to 2-day-old Sprague-Dawley rat pups as described.[7] After separation from fibroblasts, enriched cardiomyocytes were plated on 1% gelatin-coated 12-well plates for luciferase assays or on 6-cm-diameter dishes for all other experiments. Cells were grown in M199 medium containing 100 U/ml of penicillin-streptomycin and 2 mM L-glutamine without serum for 24 h before infection. HL-1 cardiac myocyte cell line was kindly provided by Dr. Claycomb (Louisiana State Univeristy Health Sciences Center).[23] Adenoviral infections were performed as previously described at a multiplicity of infection of 10 to 50 plaque forming units per ml.[7] Adβgal, AdTAK1, Ad TAB1, AdTAK1-ΔN, AdCain, AdIKKβ, AdIκBαM, and AdNFAT-luciferase reporter have been described previously.[7,8,19] AdNFκB-luciferase reporter was obtained from Vector Biolabs (Philadelphia, PA). AdshBNIP3 was kindly provided by Dr. Lorrie Kirshenbaum (University of Manitoba). In some experiments, cardiomyocytes were subjected to hypoxia in airtight chamber gassed with 95% N_2-5% CO_2 for 24 hrs. Cell viability was assessed by the Muse Count & Viability assay (Millipore). Briefly, cells were trypsinized, washed, and incubated with the Muse Count & Viability reagent, and cell viability was quantified on a Muse cell analyzer. Cell death was also measured using a Cell Meter Apoptotic and Necrotic Detection kit (ATT Bioquest, Sunnyvale, CA) according to the manufacturer's instructions.[14]

TAK1 kinase assay

TAK1 kinase assay was performed as previously described.[21] Briefly, TAK1 was immunoprecipitated with anti-TAK1 antibody (sc-7162, Santa Cruz Biotechnology) and the immunoprecipitates were incubated with GST-MKK6 (1 μg) in 25 μl of kinase assay buffer (20 mM Tris-Cl, pH 7.5, 10 mM MgCl2, 1 mM dithiothreitol, 1 mM phenylmethylsulphonyl fluoride) containing 0.3 μCi of ^{32}P-ATP for 10 min at 30°C. Samples were subjected to SDS-PAGE and visualized by PhosphorImager analysis (Amersham Pharmacia Biotech).

Western blotting

Protein extraction from mouse heart or cultured cardiomyocytes and subsequent Western blotting followed by enhanced chemiluminescence detection were performed as previously described.[14,21] The following antibodies were used: Anti-TAB2 (3745), anti-TRAF2 (4724), anti-phospho-TAK1 (Thr187; 4536), anti-phospho-NFκB p65 (sc-372), anti-IKKβ (8943), anti-Bnip3 (3769), anti-Bnip3L/Nix (12396), anti-Bax (2772), and anti-Bak (3814) were from Cell Signaling Biotechnology (Beverly, MA); Anti-TAK1 (sc-7162), anti-TAB1 (sc-6052), anti-IκBα (sc-847), Bcl-2 (sc-7382), and anti-glyceraldehyde-3-phosphate dehydrogenase (GAPDH) antibodies were from Santa Cruz Biotechnology (Santa Cruz, CA); Rabbit polyclonal anti-Rcan1 and anti-phospho-Rcan1 Ser136 were generated and purified by YenZym.[8]

Statistics

Sample size was estimated by conducting pilot experiments and power analysis. Results are presented as means ± SEM. Exact testing (Wilcoxan Rank-sum or Kruskal-Wallis test) was used for studies with small sample sizes. Data with normal distribution were also evaluated by one-way ANOVA with the Bonferroni's post hoc test or repeated measures ANOVA. The log-rank test was used for the comparison of survival data. $P<0.05$ was considered statistically significant.

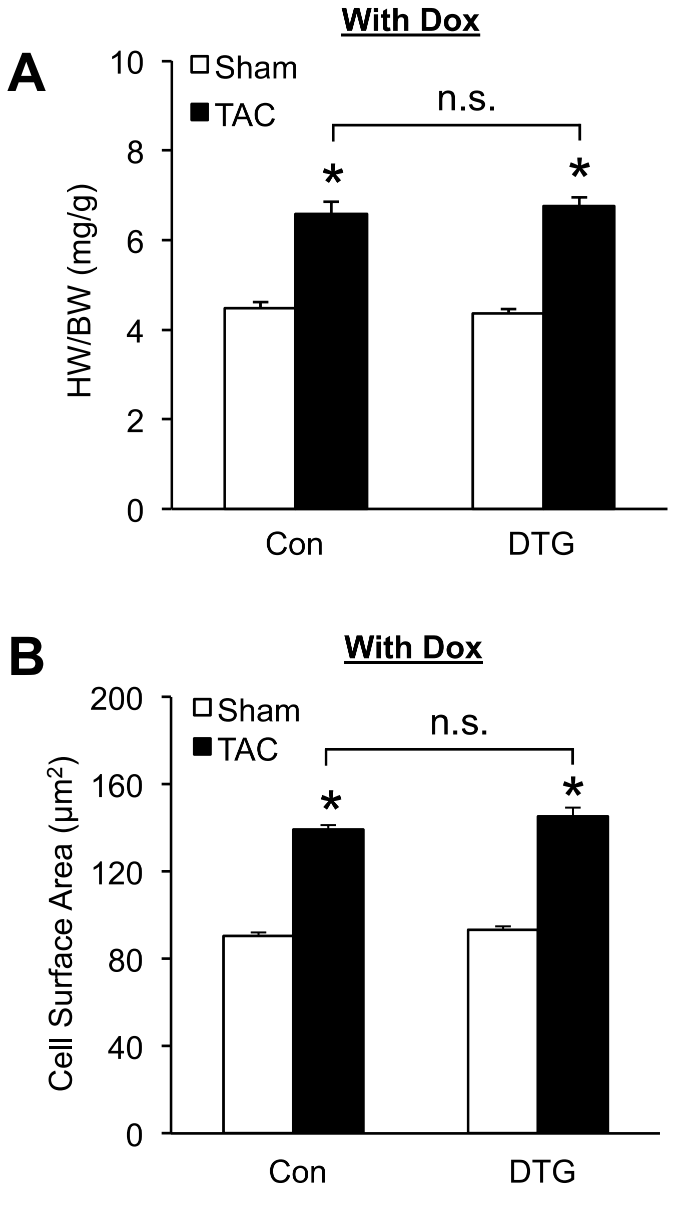

Supplemental Figure 1. TAK1ΔN DTG mice treated with Dox developed similar cardiac hypertrophy after TAC as control mice. A, Heart weight/body weight ratio (HW/BW) from control and DTG mice that were treated with Dox and then subjected to TAC or sham procedure for 2 weeks. *P<0.05 versus corresponding sham. **B,** Myocyte surface area from cardiac sections of the mice shown in A. *P<0.05 versus corresponding Sham. n.s. denotes no significance.

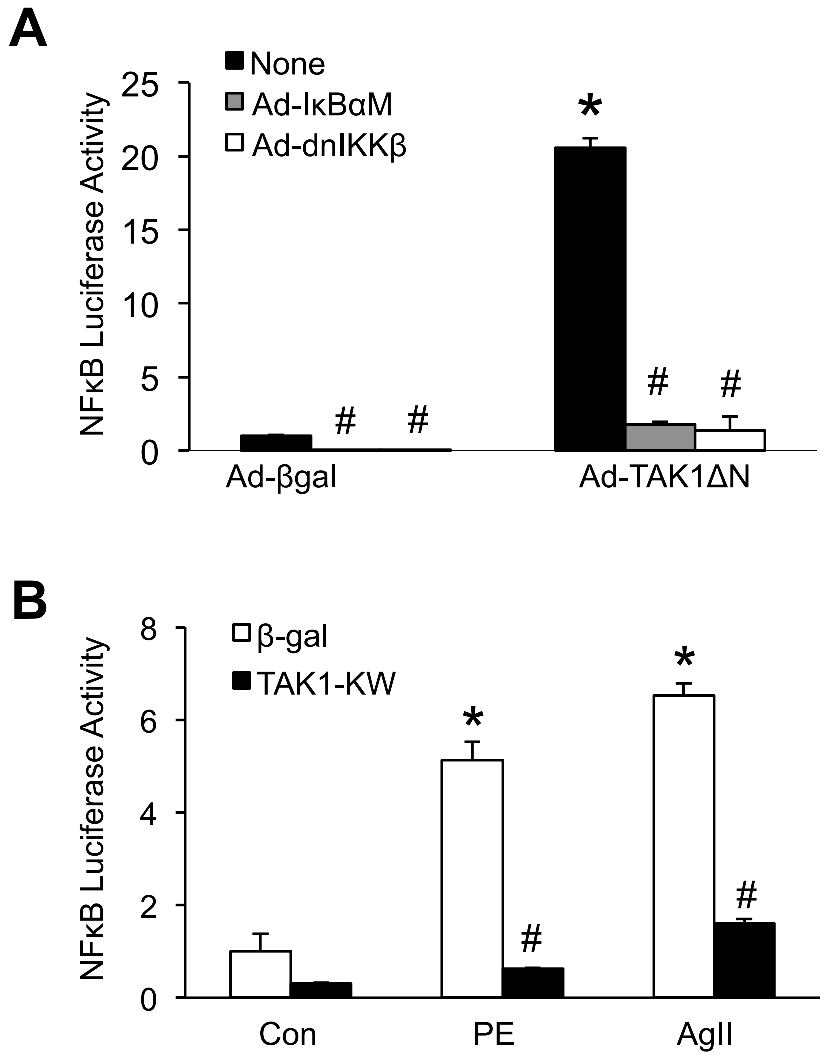

Supplemental Figure 2. TAK1 regulates NFκB transcriptional activity in cardiomyocytes. A, NFκB-luciferase activity in cardiomyocytes infected with adenoviruses expressing NFκB-luciferase reporter along with β-gal, TAK1ΔN, IκBαM or dnIKKβ. **P*<0.01 versus Ad-β-gal; #*P*<0.05 versus None in the corresponding group. **B,** NFκB-luciferase activity in cardiomyocytes infected with adenoviruses encoding NFκB-luciferase reporter along with β-gal or TAK1-KW, followed by stimulation with vehicle control (CON), phenylephrine (PE), or angiotensin II (AgII). **P*<0.01 versus β-gal Con; #*P*<0.05 versus β-gal in the corresponding group.

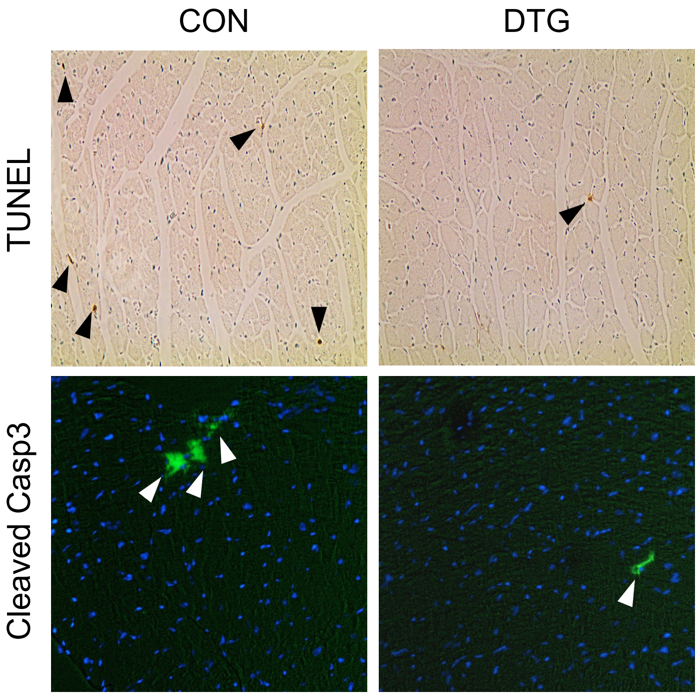

Supplemental Figure 3. TUNEL and immunohistological staining for cleaved caspase 3 in cardiac sections from control and DTG mice after 8 weeks of TAC. Black arrowheads indicate TUNEL positive cells (brown staining). White arrow heads indicate cells with cleaved caspase 3 (green staining).

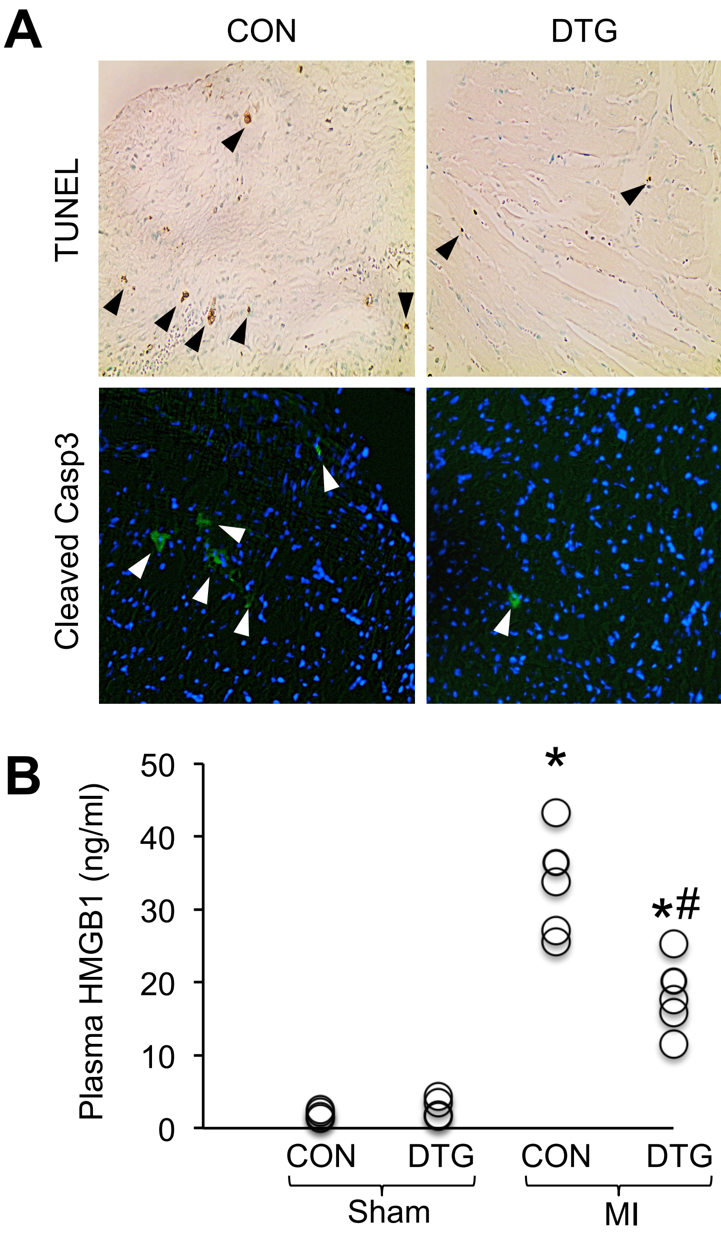

Supplemental Figure 4. TAK1 activation prevented apoptotic and necrotic cardiomyocyte death following myocardial infarction. A, TUNEL and immunohistological staining for cleaved caspase 3 in cardiac sections from control and DTG mice after 4 weeks of MI. Black arrowheads indicate TUNEL positive cells (brown staining). White arrow heads indicate cells with cleaved caspase 3 (green staining). **B,** Plasma high-mobility group box 1 (HMGB1), a biomarker for necrotic cell death and myocardial damage, from control and DTG mice after 24 h of MI or a sham surgery. *P<0.01 versus Sham; #P<0.05 versus CON MI.
